# ﻿Chromosomes of *Pseudapantelesdignus* (Muesebeck, 1938) and a review of known karyotypes of the subfamily Microgastrinae (Hymenoptera, Braconidae)

**DOI:** 10.3897/compcytogen.18.133534

**Published:** 2024-11-21

**Authors:** Vladimir E. Gokhman, María Gabriela Luna, Consuelo Vallina, María José Bressa

**Affiliations:** 1 Russian Entomological Society, Moscow, Russia Russian Entomological Society Moscow Russia; 2 Centro de Estudios Parasitológicos y de Vectores (CEPAVE, CONICET-UNLP), La Plata, Argentina Centro de Estudios Parasitológicos y de Vectores (CEPAVE, CONICET-UNLP) La Plata Argentina; 3 Universidad Nacional de San Antonio de Areco, San Antonio de Areco, Argentina Universidad Nacional de San Antonio de Areco San Antonio de Areco Argentina; 4 Grupo de Citogenética de Insectos (GCI), Instituto de Ecología, Genética y Evolución de Buenos Aires (IEGEBA), Departamento de Ecología, Genética y Evolución, Facultad de Ciencias Exactas y Naturales (FCEyN), Universidad de Buenos Aires (UBA), Buenos Aires, Argentina Universidad de Buenos Aires (UBA) Buenos Aires Argentina

**Keywords:** Base-specific fluorochromes, Braconidae, C-banding, chromosomes, fluorescence *in situ* hybridization, karyotypes, Microgastrinae, parasitoids

## Abstract

The karyotype of *Pseudapantelesdignus* (Muesebeck, 1938), an important parasitoid of a serious tomato pest *Phthorimaea* (= *Tuta*) *absoluta* Meyrick, 1917 (Lepidoptera, Gelechiidae), in the Neotropics and adjacent regions, was studied for the first time using morphometric analysis and several techniques of differential chromosome staining, i.e., C-banding and staining with base-specific fluorochromes, together with fluorescence *in situ* hybridization (FISH) with an 18S rDNA probe. We found n = 7 and 2n = 14 in *P.dignus*, with seven metacentric chromosomes of similar size in the haploid set. C-banding revealed various C-positive bands, either centromeric or interstitial, on most chromosomes. Both AT-specific and GC-specific fluorochromes, 4’6-diamidino-2-phenylindole (DAPI) and chromomycin A_3_ (CMA_3_) respectively, showed uniform staining of chromosomes. FISH visualized a single subterminal rDNA site on a medium-sized metacentric. A brief review of known chromosome sets of the subfamily Microgastrinae (Braconidae) is given; certain features of karyotype evolution of this group are discussed.

## ﻿Introduction

Parasitoid Hymenoptera are one of the most species-rich, taxonomically complicated and economically important groups of insects (Bebber et al. 2014; Forbes et al. 2018). In particular, the family Braconidae, with its high morphological and ecological diversity, contains more than 20,000 described species (Huber 2017). Moreover, Microgastrinae represent the second most speciose subfamily of Braconidae, which exceeds 3,000 described species, and up to 43,000 awaiting description, especially in the tropics (Rodriguez et al. 2013; Fernandez-Triana et al. 2020). Nevertheless, karyotypes of just a few members of this group are known so far, with only chromosomes of *Cotesiacongregata* (Say, 1836) studied using differential staining (Belle et al. 2002; Gokhman 2009). We have examined the karyotype of another species from this subfamily, *Pseudapantelesdignus* (Muesebeck, 1938), an important solitary larval endoparasitoid of a serious worldwide tomato pest *Phthorimaea* (= *Tuta*) *absoluta* Meyrick, 1917 (Lepidoptera, Gelechiidae) in the Neotropics and adjacent regions (Fernandez-Triana et al. 2014), using several techniques of differential chromosome staining. The results of this work are given below. Several biological and ecological studies have shown that *P.dignus* can potentially control *P.absoluta*, either under natural conditions or by augmentative releases in tomato fields (Salas Gervassio et al. 2019; D´Auro et al. 2021; Vallina et al. 2022). Knowledge of genetic aspects of the parasitoid life history can therefore contribute to quality mass production of biocontrol agents, and consequently, to optimization of pest control (Lommen et al. 2017). In addition to the chromosomal study of *P.dignus*, we briefly review the current state of knowledge of karyotypic diversity of Microgastrinae.

## ﻿Materials and methods

### ﻿Origin of the material studied

The laboratory stock of *P.dignus* maintained at the Centro de Estudios Parasitológicos y de Vectores (CEPAVE, CONICET and UNLP, La Plata, Argentina) originates from insects reared from cocoons of this parasitoid. These cocoons, containing pupae of *P.dignus*, were collected on tomato leaves infested with *P.absoluta* near La Plata (see Luna et al. 2007). This endoparasitoid species attacks second to fourth larval instars of gelechiid moths, particularly *P.absoluta*, depositing up to eight eggs per host during oviposition (D’Auro et al. 2021). However, only a single *P.dignus* larva survives to the third instar. At this stage, the parasitoid larva emerges from the dying host and pupates, typically spinning a silk cocoon (Luna et al. 2007). The preimaginal period lasts about 21 d; adults live ≥ 23 d in presence of the host (Vallina et al. 2022). To prepare the specimens for the chromosomal study, twenty cohorts of *P.dignus* were initiated by exposing a two-day-old, fertilized female to twenty larvae of *P.absoluta* inside leaf mines. The larvae were then kept in one-liter plastic containers and fed with 50% honey syrup ad libitum. Cohorts were generated sequentially to synchronize the rearing process and to obtain material for dissections at the correct developmental stage. All cultures were maintained at 25 °C and 60 to 75% humidity, with a 14 h light: 10 h dark photoperiod in a walk-in environmental chamber. Voucher specimens from this study are deposited at CEPAVE (La Plata, Argentina).

### ﻿Preparation and staining of chromosomes

Chromosomal preparations were obtained from cerebral ganglia of parasitoid prepupae generally following the protocol developed by Imai et al. (1988) with certain modifications (see, e.g., Gokhman et al. 2019). Ganglia were extracted from insects dissected in 0.5% hypotonic sodium citrate dihydrate solution containing 0.005% colchicine. The extracted ganglia were then transferred to fresh hypotonic solution and incubated for 30 min at room temperature. The material was transferred onto a pre-cleaned microscope slide using a Pasteur pipette and then gently flushed with Fixative I (glacial acetic acid: absolute ethanol: distilled water 3:3:4). The tissues were disrupted using dissecting needles in an additional drop of Fixative I. A drop of Fixative II (glacial acetic acid: absolute ethanol 1:1) was applied to the center of the area, and the more aqueous phase was blotted off the edges of the slide. The slides were dried for approximately 30 min and stored at room temperature.

For routine staining, chromosome preparations were stained overnight using a freshly prepared 3% Giemsa solution (Merck KGaA, Darmstadt, Germany). C-banding and sequential staining with AT-specific 4’,6-diamidino-2-phenylindole (DAPI; Fluka BioChemika, Sigma Aldrich Production GmbH, Buchs, Switzerland) and GC-specific chromomycin A_3_ (CMA_3_; Fluka BioChemika) were carried out following Poggio et al. (2011). For C-banding, the pre-treated slides were stained with DAPI to improve the resolution of C-bands (Barros e Silva and Guerra 2010; Poggio et al. 2011).

Unlabeled 18S ribosomal DNA (rDNA) probe was generated by polymerase chain reaction (PCR) using universal arthropod primers: forward 5’-CCTGAGAAACGGCTACCACATC-3’ and reverse 5’-GAGTCTCGTTCGTTATCGGA-3’ (Whiting 2002). Total genomic DNA of *Dysdercusalbofasciatus* Berg, 1878 (Hemiptera, Pyrrhocoridae), obtained by standard phenol-chloroform-isoamyl alcohol extraction, was used as a template. PCR was performed following the procedure described by Fuková et al. (2005) and Bressa et al. (2009). The PCR product displayed a single band of approximately 1,000 bp on a 1% agarose gel. The band was cut out from the gel, and the DNA was extracted using a QIAquick Gel Extraction Kit (Qiagen GmbH, Hilden, Germany). The 18S rDNA fragment was re-amplified by PCR and subsequently labeled with biotin-14-dUTP by nick translation using a BioNick Labeling System (Invitrogen, Life Technologies Inc., San Diego, CA, USA). FISH with biotinylated 18S rDNA probe was performed following the procedure developed by Sahara et al. (1999) with several modifications described by Fuková et al. (2005) and Bressa et al. (2009).

### ﻿Image acquisition and analysis

Metaphase plates of *P.dignus* were examined and photographed with an optical microscope Zeiss Axioskop 40 FL fitted with a digital color camera Axiocam 208 (Carl Zeiss, Germany) as well as an epifluorescence microscope Leica DMLB fitted with a digital camera Leica DFC350 FX CCD (Leica Microsystems Imaging Solutions Ltd., Cambridge, UK) respectively. To produce illustrations, the resulting images were processed with the image processing programs ZEN version 3.0 (blue edition), Leica IM50 version 4.0, Adobe Photoshop CC version 14.0, and GIMP version 2.10. Black-and-white images of chromosomes were captured separately for each fluorescent dye. Images were pseudocolorized (light blue, green, and red for DAPI, CMA_3_, and Cy3, respectively) and processed with Adobe Photoshop CC version 14.0. KaryoType version 2.0 software (Altınordu et al. 2016) was also used for taking measurements from ten haploid metaphase plates of *P.dignus*. The chromosomes were classified following guidelines provided by Levan et al. (1964). All studies were conducted at CEPAVE (La Plata, Argentina), IEGEBA/DEGE of FCEyN of Universidad de Buenos Aires (Ciudad Autónoma de Buenos Aires, Argentina), and the Botanical Garden of Moscow State University (Moscow, Russia).

## ﻿Results

**Figure 1.**
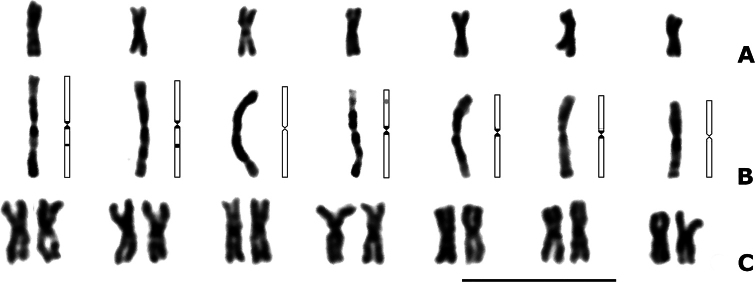
Karyograms of Giemsa-stained chromosomes of *P.dignus***A** haploid, metaphase **B** haploid, prometaphase **C** diploid, metaphase. For **B** idiogram for each chromosome demonstrating heterochromatin distribution and position of the secondary constriction in black and grey respectively, is also shown. Scale bar: 10 μm.

The haploid karyotype of *P.dignus* contains seven metacentric chromosomes, which exhibit a gradual decrease in size (n = 7; Fig. 1A, B, Table 1). In prometaphase chromosomes, pericentromeric and interstitial heterochromatic segments are visible. In addition, a distinct secondary constriction is visible on a medium-sized chromosome (Fig. 1B). The diploid chromosome set of *P.dignus* consists of seven pairs of similar metacentric chromosomes (Fig. 1C; 2n = 14). Chromosome relative lengths of the haploid set (RLs) range from 16.15 ± 0.44 per cent for the longest chromosome to 12.35 ± 0.46 per cent for the smallest one (Table 1). Despite some difference in RLs between the longest and the shortest chromosome, the karyotype of *P.dignus* is fairly homogeneous in chromosome morphology and size, suggesting that this species possesses a highly symmetrical karyotype (Stebbins 1950).

**Table 1.** Relative lengths (RLs) and centromeric indices (CIs) of chromosomes of *P.dignus* (mean ± SD).

Chr. no.	RL, per cent	CI, per cent
1	16.15 ± 0.44	48.62 ± 0.86
2	15.42 ± 0.36	47.91 ± 2.68
3	14.67 ± 0.40	46.13 ± 2.74
4	14.35 ± 0.38	47.60 ± 1.71
5	13.87 ± 0.35	47.41 ± 2.03
6	13.19 ± 0.43	47.07 ± 2.38
7	12.35 ± 0.46	46.39 ± 2.43

**Figure 2.**
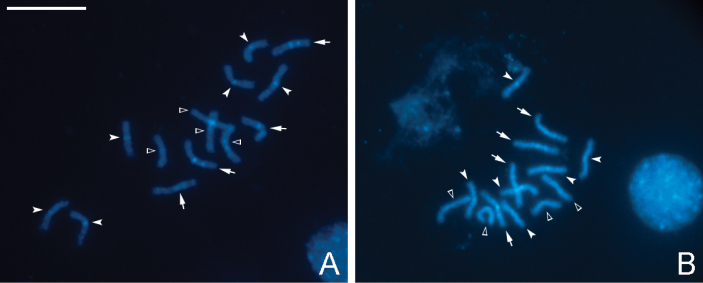
C-banded and DAPI-stained diploid metaphase plates of *P.dignus* (**A, B**). Arrows indicate chromosomes with both centromeric and interstitial C-positive bands, filled arrowheads indicate chromosomes with only centromeric C-positive bands, and empty arrowheads indicate lack of C-positive bands respectively. Scale bar: 10 μm.

**Figure 3.**
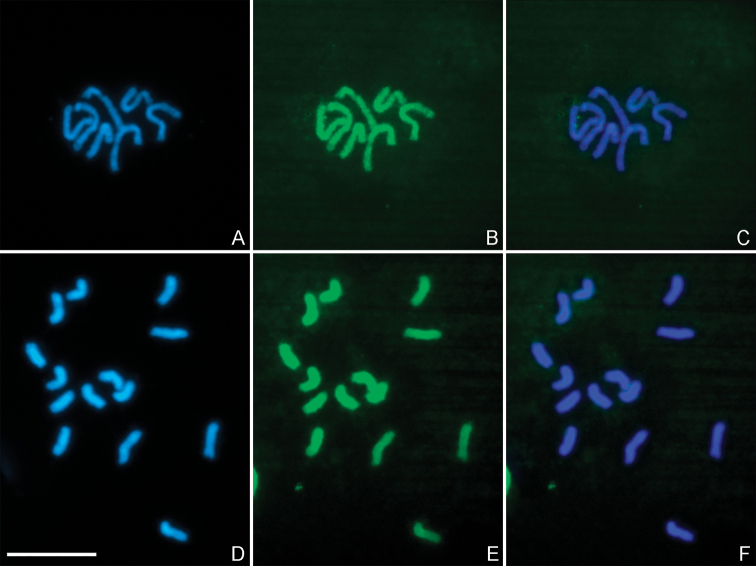
DAPI/CMA_3_-stained metaphase haploid (**A–C**) and diploid (**D–F**) plates of *P.dignus***A, D**DAPI staining **B, E** CMA_3_ staining **C, F** merged images. Scale bar: 10 μm.

C-banding reveals different patterns in the amount and location of constitutive heterochromatin on the chromosomes of *P.dignus*. Specifically, three pairs of chromosomes in the diploid set exhibit only centromeric C-positive bands. These bands are brighter and more conspicuous on chromosomes of two of the pairs than on the third one. On chromosomes of the two other pairs, strong C-positive centromeric bands are accompanied by small interstitial ones. No C-bands are detected on the remaining chromosomes (Fig. 2A, B). All mitotic chromosomes show relatively uniform fluorochrome staining with both DAPI and CMA_3_ (Fig. 3).

**Figure 4.**
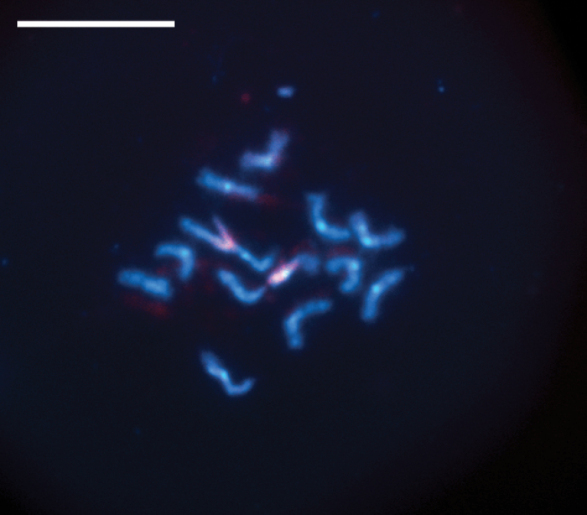
FISH with 18S rDNA probe on chromosomes of the diploid karyotype of *P.dignus*. Probe signals are indicated in red. Scale bar: 10 μm.

In the diploid karyotype of *P.dignus*, FISH with an 18S rDNA probe reveals a single subterminal rDNA cluster on a pair of medium-sized metacentric chromosomes (Fig. 4). The location of the rDNA cluster apparently co-localizes with the secondary constriction observed on a specific medium-sized chromosome of this species (Fig. 1B).

## ﻿Discussion

The karyotype of *P.dignus* is characterized by the lowest chromosome number found in the subfamily Microgastrinae, with n values for other species ranging from 9 to 11 (Table 2). Moreover, members of this group exhibit considerable diversity in terms of chromosomal morphology. Specifically, karyotypes of most studied species of Microgastrinae, including *P.dignus*, *Cotesiaglomerata* (Linnaeus, 1758) and *C.congregata*, predominantly contain biarmed chromosomes (Belle et al. 2002; Zhou et al. 2006). On the other hand, the chromosome set of “*Apanteles* sp.” mostly harbors subtelocentric and/or acrocentric chromosomes (Hoshiba and Imai 1993). According to our phylogenetic reconstruction of karyotype evolution in Braconidae (Gokhman 2009), n values of 9 to 11 also prevail in the non-cyclostome lineage of this family; thus, the lower chromosome number, n = 7 found in *P.dignus*, is apparently derived. This lends further support from the suggested basal position of the genus *Microplitis* Förster, 1862 (Quicke 2015 and references therein), with n = 10–11 (Table 2).

**Table 2.** Chromosome numbers of parasitoids of the subfamily Microgastrinae.

Species	n(2n)	Reference
*Apanteles* sp.^†^	11	Hoshiba and Imai 1993
*Cotesiacongregata* (Say, 1836)	10	Belle et al. 2002
*C.glomerata* (Linnaeus, 1758)	10(20)	Zhou et al. 2006
*Microgasterluctuosa* Haliday, 1834 (= *curvicrus* Thomson, 1895)	(18)	Gokhman 2004
*Microplitisdemolitor* Wilkinson, 1934	10	M. Strand, pers. comm., cited in: Gokhman 2009
*M.ratzeburgii* (Ruthe, 1858)	(22)	Gokhman 2009
*M.tuberculifer* (Wesmael, 1837)	(22)	Gokhman 2009
*Pseudapantelesdignus* (Muesebeck, 1938)	7(14)	Present paper

^†^Like many other identifications in the cited work, this one is dubious and may well refer to any other member of Microgastrinae, e.g., *Microplitis* sp.

Up to now, only three species of the family Braconidae have been studied using C-banding, “*Apanteles* sp.” (Hoshiba and Imai 1993), *Aphidiuservi* Haliday, 1834 (n = 5, 2n = 10 and 12) (Gokhman and Westendorff 2003), and *Diachasmimorphalongicaudata* (Ashmead, 1905) (n = 20, 2n = 40) (Carabajal Paladino et al. 2013). In “*Apanteles* sp.”, the karyotype consists of a single pseudoacrocentric chromosome with the fully heterochromatic shorter arm, along with nine more or less euchromatic subtelocentrics/acrocentrics together with an apparently euchromatic submetacentric chromosome (Hoshiba and Imai 1993). Chromosomes of *A.ervi* are also predominantly euchromatic; however, a few studied females carried an additional pair of almost fully heterochromatic acrocentric chromosomes (Gokhman and Westendorff 2003). In *D.longicaudata*, most chromosomes are pseudoacrocentric, and many of them carry large segments of pericentromeric heterochromatin (Carabajal Paladino et al. 2013). Our results obtained using C-banding in *P.dignus* thus revealed differences in the size and location of heterochromatic segments compared to other Braconidae. Moreover, three different C-banding patterns were found within the diploid chromosome set of *P.dignus*, resulting in a species-specific distribution of constitutive heterochromatin. Various mechanisms have been proposed to account for the variation in the content and distribution of heterochromatin both within and between species, e.g., multiple replication, unequal exchanges, accumulation or elimination [reviewed by John (1988)], which could explain the differences observed among the four members of this family with known heterochromatin distribution.

Previously, multiple rDNA loci per haploid karyotype have been detected in certain Hymenoptera species using Ag-NOR, DAPI/CMA_3_-banding and/or FISH; however, typically, only a single rDNA cluster is active (Hirai et al. 1994; Matsumoto et al. 2002; Gokhman 2009; Carabajal Paladino et al. 2013). In this order, as well as in other insects, CMA_3_-positive bands co-localize with nucleolus organizing regions (NORs), suggesting that rDNA clusters are typically rich in GC base pairs (Camacho et al. 1991; Hirai et al. 1994; Vitturi et al. 1999; Maffei et al. 2001; Costa et al. 2004; Papeschi and Bressa 2006; Bolsheva et al. 2012; Gokhman et al. 2016; Gokhman and Kuznetsova 2024). However, in the parasitoid *D.longicaudata* (Carabajal Paladino 2011) and in certain true bugs (Hemiptera, Heteroptera) (Bressa et al. 2005; Severi-Aguiar and de Azeredo-Oliveira 2005; Morielle-Souza and Azeredo-Oliveira 2007; Poggio 2012), no such association has been demonstrated. The results of DAPI/CMA_3_-banding indicate that all chromosomes of *P.dignus* lack specific regions enriched either in AT or GC base pairs. Thus, the NOR of *P.dignus* is apparently not associated with CMA_3_-positive chromosomal segments as well.

To date, the only karyotypic study of Microgastrinae involving *in situ* hybridization was performed on *C.congregata* (Belle et al. 2002). Specifically, this technique visualized a single cluster of rDNA as well as certain DNA sequences coding for a symbiotic polydnavirus. In the haploid karyotype of this species, both sites appeared to have subterminal localization on shorter arms of the two different subtelocentric chromosomes (Belle et al. 2002). Contrary to *C.congregata*, all chromosomes of *P.dignus* are metacentric, but the single NOR is also located subterminally on a particular chromosome of the latter species. Interestingly, six rDNA clusters per haploid karyotype were earlier discovered in *D.longicaudata*, but, nevertheless, they all also have subterminal localization on chromosomes (Carabajal Paladino et al. 2013).

Most NORs in eukaryotic genomes are located in heterochromatic regions (Goessens 1984; Hadjiolov 1985; Babu and Verma 1987; Gokhman and Kuznetsova 2024), likely because certain heterochromatin-associated genes can silence repetitive DNA sequences and suppress recombination among them (Gottlieb and Esposito 1989). Based on the length and morphology of the chromosomes of *P.dignus*, we conclude that rDNA clusters in this species are located within the C-positive interstitial bands of one of the two chromosome pairs that carry these bands (Fig. 1B; see above). A similar pattern was previously observed in *D.longicaudata* (Carabajal Paladino et al. 2013). In this parasitoid species, hybridization signals with the 18S rDNA probe were also detected in heterochromatic regions. Nevertheless, both these regions and the rDNA clusters were CMA_3_-negative and, consequently, not enriched in GC base pairs.

Although currently no karyotypically distinct groups of cryptic species of Microgastrinae are known, this situation may change as an increasing number of members of this subfamily are examined, similarly to other taxa of parasitic wasps (Gokhman 2009, 2022). Moreover, chromosomal analysis of Microgastrinae will provide us further insights into their genetic features, which can, in turn, offer important information necessary for mass rearing and other aspects of applied use of these parasitoids. In addition, the results of the karyotypic study of this subfamily are already being used to verify the results of chromosome-level genome assemblies (Gokhman 2022, 2023). For example, this includes *C.congregata* and *C.glomerata* (Gauthier et al. 2021; Pinto et al. 2021). Furthermore, both genome assemblies of *Microplitismanilae* Ashmead, 1904 suggest n = 11 for this parasitoid (Shu et al. 2023; Yan et al. 2023). Since we report the same n value for two other *Microplitis* species (Table 2), these results appear plausible.

## ﻿Author contributions

VEG: conceptualization, data curation, formal analysis, investigation, methodology, supervision, validation, visualization, writing – original draft, writing – review and editing. MGL: data curation, funding acquisition, methodology, resources, validation, writing – review and editing. CV: methodology, resources, writing – review and editing. MJB: conceptualization, data curation, formal analysis, investigation, methodology, resources, validation, visualization, writing – review and editing. All authors have read and agreed with the final version of the manuscript.

## References

[B1] Altınordu F Peruzzi L Yu Y He X (2016) A tool for the analysis of chromosomes: KaryoType. Taxon 65(3): 586–592. 10.12705/653.9

[B2] Babu KA Verma RS (1987) Chromosome structure: Euchromatin and heterochromatin. International Review of Cytology 108: 1–60. 10.1016/s0074-7696(08)61435-7 2822591

[B3] Barros e Silva AE Guerra M (2010) The meaning of DAPI bands observed after C-banding and FISH procedures. Biotechnic and Histochemistry 85: 115–125. 10.3109/10520290903149596 19657781

[B4] Bebber DP Polaszek A Wood JRI Barker C Scotland RW (2014) Taxonomic capacity and author inflation. New Phytologist 202: 741–742. 10.1111/nph.12745 24716516

[B5] Belle E Beckage NE Rousselet J Poirié M Lemeunier F Drezen J-M (2002) Visualization of polydnavirus sequences in a parasitoid wasp chromosome. Journal of Virology 76(11): 5793–5796. 10.1128/jvi.76.11.5793-5796.2002 11992007 PMC137038

[B6] Bolsheva NL Gokhman VE Muravenko OV Gumovsky AV Zelenin AV (2012) Comparative cytogenetic study on two species of the genus *Entedon* Dalman, 1820 (Hymenoptera: Eulophidae) using DNA-binding fluorochromes and molecular and immunofluorescent markers. Comparative Cytogenetics 6(1): 79–92. 10.3897/compcytogen.v6i1.2349 24260653 PMC3833767

[B7] Bressa MJ Larramendy M Papeschi AG (2005) Heterochromatin characterization in five species of Heteroptera. Genetica 124: 307–317. 10.1007/s10709-005-4524-3 16134342

[B8] Bressa MJ Papeschi AG Vitková M Kubičkova S Fuková I Pigozzi MI Marec F (2009) Sex chromosome evolution in cotton stainers of the genus *Dysdercus* (Heteroptera: Pyrrhocoridae). Cytogenetics and Genome Research 125: 292–305. 10.1159/000235936 19864893

[B9] Camacho JPM Cabrero J Viseras E Lopez-León MD Navas-Castillo J Alche JP (1991) G banding in two species of grasshoppers and its relationships to C, N and fluorescence banding techniques. Genome 34: 638–643. 10.1139/g91-097

[B10] Carabajal Paladino LZ (2011) Genética y citogenética de la determinación del sexo en *Diachasmimorphalongicaudata* (Hymenoptera, Braconidae). PhD thesis. Universidad de Buenos Aires, Facultad de Ciencias Exactas y Naturales, 111 pp. [In Spanish] https://bibliotecadigital.exactas.uba.ar/download/tesis/tesis_n5050_CarabajalPaladino.pdf

[B11] Carabajal Paladino LZ Papeschi AG Lanzavecchia S Cladera JL Bressa MJ (2013) Cytogenetic characterization of *Diachasmimorphalongicaudata* (Hymenoptera: Braconidae), a parasitoid wasp used as a biological control agent. European Journal of Entomology 110: 401–409. https://www.eje.cz/pdfs/110/3/401

[B12] Costa KF Brito RM Miyazawa CS (2004) Karyotypic description of four species of *Trigona* (Jurine, 1807) (Hymenoptera, Apidae, Meliponini) from the State of Mato Grosso, Brazil. Genetics and Molecular Biology 27: 187–190. 10.1590/s1415-47572004000200010

[B13] D’Auro F Luna MG Liljesthröm GG (2021) Functional response and egg distribution among hosts by *Pseudapantelesdignus*, a larval endoparasitoid of *Tutaabsoluta*. Journal of Applied Entomology 145: 688–696. 10.1111/jen.12889

[B14] Fernández-Triana JL Janzen DH Hallwachs W Whitfield JB Smith MA Kula R (2014) Revision of the genus *Pseudapanteles* (Hymenoptera, Braconidae, Microgastrinae), with emphasis on the species in Area de Conservación Guanacaste, northwestern Costa Rica. ZooKeys 446: 1–82. 10.3897/zookeys.446.8195 PMC4205727 25349512

[B15] Fernandez-Triana J Shaw MR Boudreault C Beaudin M Broad GR (2020) Annotated and illustrated world checklist of Microgastrinae parasitoid wasps (Hymenoptera, Braconidae). ZooKeys 920: 1–1089. 10.3897/zookeys.920.39128 32390740 PMC7197271

[B16] Forbes AA Bagley RK Beer MA Hippee AC Widmayer HA (2018) Quantifying the unquantifiable: Why Hymenoptera, not Coleoptera, is the most speciose animal order. BMC Ecology 18: 21. 10.1186/s12898-018-0176-x PMC6042248 30001194

[B17] Fuková I Nguyen P Marec F (2005) Codling moth cytogenetics: karyotype, chromosomal location of rDNA, and molecular differentiation of sex chromosomes. Genome 48: 1083–1092. 10.1139/g05-063 16391677

[B18] Gauthier J Boulain H van Vugt JJFA Baudry L Persyn E Aury J-M Noel B Bretaudeau A Legeai F Warris S Chebbi MA Dubreuil G Duvic B Kremer N Gayral P Musset K Josse T Bigot D Bressac C Moreau S Periquet G Harry M Montagné N Boulogne I Sabeti-Azad M Maïbèche M Chertemps T Hilliou F Siaussat D Amselem J Luyten I Capdevielle-Dulac C Labadie K Merlin BL Barbe V de Boer JG Marbouty M Cônsoli FL Dupas S Hua-Van A Goff GL Bézier A Jacquin-Joly E Whitfield JB Vet LEM Smid HM Kaiser L Koszul R Huguet E Herniou EA Drezen J-M (2021) Chromosomal scale assembly of parasitic wasp genome reveals symbiotic virus colonization. Communications Biology 4: 104. 10.1038/s42003-020-01623-8 PMC7822920 33483589

[B19] Gokhman VE (2004) Chromosomes of the family Braconidae. Proceedings of the Russian Entomological Society 75(1): 96–101. [In Russian]

[B20] Gokhman VE (2009) Karyotypes of Parasitic Hymenoptera. Dordrecht, Springer, [XIII +] 183 pp. 10.1007/978-1-4020-9807-9

[B21] Gokhman VE (2022) Comparative karyotype analysis of parasitoid Hymenoptera (Insecta): major approaches, techniques, and results. Genes 13: 751. 10.3390/genes13050751 PMC9141968 35627136

[B22] Gokhman VE (2023) Chromosome study of the Hymenoptera (Insecta): from cytogenetics to cytogenomics. Comparative Cytogenetics 17: 239–250. 10.3897/compcytogen.17.112332 37953851 PMC10632776

[B23] Gokhman VE Kuznetsova VG (2024) Structure and evolution of ribosomal genes of insect chromosomes. Insects 15: 593. 10.3390/insects15080593 PMC11354594 39194798

[B24] Gokhman VE Westendorff (2003) Chromosomes of *Aphidiuservi* Haliday, 1834 (Hymenoptera, Braconidae). Beiträge zur Entomologie 53(1): 161–165. 10.21248/contrib.entomol.53.1.161-165

[B25] Gokhman VE Bolsheva NL Govind S Muravenko OV (2016) A comparative cytogenetic study of *Drosophila* parasitoids (Hymenoptera, Figitidae) using DNA-binding fluorochromes and FISH with 45S rDNA probe. Genetica 144: 335–339. 10.1007/s10709-016-9902-5 27150102

[B26] Gokhman VE Cioffi MB König C Pollmann M Gantert C Krogmann L Steidle JLM Kosyakova N Liehr T Al-Rikabi A (2019) Microdissection and whole chromosome painting confirm karyotype transformation in cryptic species of the *Lariophagusdistinguendus* (Förster, 1841) complex (Hymenoptera: Pteromalidae). PLoS ONE 14(11): e0225257. 10.1371/journal.pone.0225257 PMC6855445 31725808

[B27] Goessens G (1984) Nucleolar structure. International Review of Cytology 84: 107–158. 10.1016/s0074-7696(08)62441-9 6201455

[B28] Gottlieb S Esposito RE (1989) A new role for a yeast transcriptional silencer gene, SIR2, in regulation of recombination in ribosomal DNA. Cell 56: 771–776. 10.1016/0092-8674(89)90681-8 2647300

[B29] Hadjiolov AA (1985) The nucleolus and ribosome biogenesis. Wien, Springer-Verlag, XII + 272 p. 10.1007/978-3-7091-8742-5

[B30] Hirai H Yamamoto M-T Ogura K Satta Y Yamada M Taylor RW Imai HT (1994) Multiplication of 28S rDNA and NOR activity in chromosome evolution among ants of the *Myrmeciapilosula* species complex. Chromosoma 103: 171–178. 10.1007/bf00368009 7924619

[B31] Hoshiba H Imai HT (1993) Chromosome evolution of bees and wasps (Hymenoptera, Apocrita) on the basis of C-banding pattern analyses. Japanese Journal of Entomology 61(3): 465–492.

[B32] Huber JT (2017) Biodiversity of Hymenoptera. In: Foottit RG Adler PH (Eds) Insect Biodiversity: Science and Society.2^nd^ edn. Oxford, Wiley Blackwell, 419–461. 10.1002/9781118945568.ch12

[B33] Imai HT Taylor RW Crosland MWJ Crozier RH (1988) Modes of spontaneous chromosomal mutation and karyotype evolution in ants with reference to the minimum interaction hypothesis. Japanese Journal of Genetics 63: 159–185. 10.1266/jjg.63.159 3273765

[B34] John B (1988) The biology of heterochromatin. In: Verma RS (Ed.) Heterochromatin: Molecular and Structural Aspects.Cambridge, Cambridge University Press, 1–128.

[B35] Levan A Fredga K Sandberg AA (1964) Nomenclature for centromeric position on chromosomes. Hereditas 52: 201–220. 10.1111/j.1601-5223.1964.tb01953.x

[B36] Lommen STE de Jong PW Pannebakker BA (2017) It is time to bridge the gap between exploring and exploiting: prospects for utilizing intraspecific genetic variation to optimize arthropods for augmentative pest control – a review. Entomologia Experimentalis et Applicata 162: 108–123. 10.1111/eea.12510

[B37] Luna MG Sánchez NE Pereyra PC (2007) Parasitism of *Tutaabsoluta* (Lepidoptera, Gelechiidae) by *Pseudapantelesdignus* (Hymenoptera, Braconidae) under laboratory conditions. Environmental Entomology 36(4): 887–893. 10.1093/ee/36.4.887 17716480

[B38] Maffei EMD Pompolo SG Campos LAO Petitpierre E (2001) Sequential FISH analysis with rDNA genes and Ag-NOR banding in the lady beetle *Ollav-nigrum* (Coleoptera: Coccinellidae). Hereditas 135: 13–18. 10.1111/j.1601-5223.2001.00013.x 12043703

[B39] Matsumoto K Yamamoto DS Sumitani M Lee JM Hatakeyama M Oishi K (2002) Detection of single copy gene on a mitotic metaphase chromosome by fluorescent in situ hybridization (FISH) in the sawfly *Athaliarosae*. Archives on Insect Biochemistry and Physiology 41: 34–40. 10.1002/arch.10005 11754092

[B40] Morielle-Souza A Azeredo-Oliveira MTV (2007) Differential characterization of holocentric chromosomes in triatomines (Heteroptera, Triatominae) using different staining techniques and fluorescent *in situ* hybridization. Genetics and Molecular Research 6: 713–720. 18050092

[B41] Papeschi AG Bressa MJ (2006) Evolutionary cytogenetics in Heteroptera. Journal of Biological Research 5: 3–21.

[B42] Pinto BJ Weis JJ Gamble T Ode PJ Paul R Zaspel JM (2021) A chromosome-level genome assembly of the parasitoid wasp, *Cotesiaglomerata* (Hymenoptera: Braconidae). Journal of Heredity 112(6): 558–564. 10.1093/jhered/esab032 34043785

[B43] Poggio MG (2012) Aportes al conocimiento de los cromosomas holocíneticos de Hemiptera: estudios citogenéticos y evolutivos en especies de Cimicomorpha. PhD thesis, Universidad de Buenos Aires, Facultad de Ciencias Exactas y Naturales, 195 pp. [In Spanish] https://bibliotecadigital.exactas.uba.ar/download/tesis/tesis_n5219_Poggio.pdf

[B44] Poggio MG Bressa MJ Papeschi AG (2011) Male meiosis, heterochromatin characterization and chromosomal location of rDNA in *Microtomuslunifer* (Berg, 1900) (Hemiptera: Reduviidae: Hammacerinae). Comparative Cytogenetics 5: 1–22. 10.3897/compcytogen.v5i1.1143 24260616 PMC3833732

[B45] Quicke DLJ (2015) The Braconid and Ichneumonid Parasitoid Wasps: Biology, Systematics, Evolution and Ecology. 1^st^ edn. Oxford etc., John Wiley & Sons, [XV +] 681 pp. 10.1002/9781118907085

[B46] Rodriguez JJ Fernández-Triana J Smith MA Janzen DH Hallwachs W Erwin T Whitfield JB (2013) Extrapolations from field studies and known faunas converge on dramatically increased estimates of global microgastrine parasitoid wasp species richness (Hymenoptera: Braconidae). Insect Conservation and Diversity 6(4): 530–536. 10.1111/icad.12003

[B47] Sahara K Marec F Traut W (1999) TTAGG telomeric repeats in chromosomes of some insects and other arthropods. Chromosome Research 7: 449–460. 10.1023/A:1009297729547 10560968

[B48] Salas Gervassio NG Luna MG Minardi GM Sánchez NE (2019) Assessing inoculative releases of *Pseudapantelesdignus* (Hymenoptera: Braconidae) for the biological control of *Tutaabsoluta* (Lepidoptera: Gelechiidae). Crop Protection 124: 104830. 10.1016/j.cropro.2019.05.024

[B49] Severi-Aguiar GDC de Azeredo-Oliveira MTV (2005) Localization of rDNA sites in holocentric chromosomes of three species of triatomines (Heteroptera, Triatominae). Genetics and Molecular Research 4(4): 704–709. 16475115

[B50] Shu X Yuan R Zheng B Wang Z Ye X Tang P Chen X (2023) Chromosome-level genome assembly of *Microplitismanilae* Ashmead, 1904 (Hymenoptera: Braconidae). Scientific Data 10: 266. 10.1038/s41597-023-02190-3 PMC10172384 37164995

[B51] Stebbins GL (1950) Variation and Evolution in Plants. New York, Columbia University Press, 643 pp. 10.7312/steb94536

[B52] Vallina C Greco NM D´Auro F Sánchez NE Luna MG (2022) Biological parameters of three Argentinian populations of *Pseudapantelesdignus*, a larval endoparasitoid of *Tutaabsoluta*, at various temperatures. Biological Control 165: 104791. 10.1016/j.biocontrol.2021.104791

[B53] Vitturi R Colomba MS Barbieri R Zunino M (1999) Ribosomal DNA location in the scarab beetle *Thorectesintermedius* (Costa) (Coleoptera: Geotrupidae) using banding and fluorescent *in-situ* hybridization. Chromosome Research 7: 255–260. 10.1023/a:1009270613012 10461870

[B54] Whiting MF (2002) High-throughput DNA sequencing for systematics. In: DeSalle R Giribet G Wheeler W (Eds) Techniques in Molecular Systematics and Evolution.Methods and Tools in Biosciences and Medicine. Basel, Birkhäuser, 328–350. 10.1007/978-3-0348-8125-8_15

[B55] Yan B Di X Yang M Wu H Yu X Zhang F (2023) Chromosome-scale genome assembly of the solitary parasitoid wasp *Microplitismanilae* Ashmead, 1904 (Braconidae: Microgastrinae). Genome Biology and Evolution 15(8): evad144. 10.1093/gbe/evad144 PMC10448859 37515590

[B56] Zhou Y Gu H Dorn S (2006) Single-locus sex determination in the parasitoid wasp *Cotesiaglomerata* (Hymenoptera: Braconidae). Heredity 96: 487–492. 10.1038/sj.hdy.6800829 16622470

